# HOW GOAL REPRESENTATIONS MODULATE ASSOCIATIONS BETWEEN EVERYDAY AFFECT AND GOAL PURSUIT AMONG OLDER ADULTS

**DOI:** 10.1093/geroni/igac059.2402

**Published:** 2022-12-20

**Authors:** Yoonseok Choi, Denis Gerstorf, Maureen Ashe, Kenneth Madden, Christiane Hoppmann

**Affiliations:** University of British Columbia, Vancouver, British Columbia, Canada; Humboldt Universität zu Berlin, Berlin, Berlin, Germany; The University of British Columbia, Vancouver, British Columbia, Canada; University of British Columbia, Vancouver, British Columbia, Canada; University of British Columbia, Vancouver, British Columbia, Canada

## Abstract

Individuals differ in the extent to which they represent their goals as hoped-for versus feared states. We examined the role of such goal representations for how everyday affective experiences and goal pursuit are intertwined. When goals are represented as hoped-for states, we expected stronger associations between daily positive affect and goal pursuit. In contrast, when goals are represented as feared states, we expected stronger associations between daily negative affect (particularly fear) and goal pursuit. We used seven days of repeated daily life assessments from 238 older individuals (Age: M = 70.5 years, SD = 5.99, 59-87 years; N = 119 couples). At baseline, participants reported three goals they planned to pursue over the study period and the extent to which each goal referred to something they hoped-for or feared. During the daily life assessments, participants reported their current affective states and momentary goal pursuit (goal engagement and goal progress) three times per day (11 AM, 4 PM, 9 PM). Multilevel analyses regarding participants’ most salient goal provide initial evidence supporting the expected interactions of goal representations on everyday affect–goal pursuit links. Specifically, individuals with a strong hope-focus in their goals engaged in more goal pursuit when positive affect was up than individuals whose goals were low in hope-focus. In contrast, those with feared goals engaged in more goal pursuit when reporting increased fear. Findings are discussed in the context of the possible selves literature. Future analyses will examine lead-lag effects to address the temporal order underlying affect-goal pursuit associations.

